# Changes in urinary risk profile after short-term low sodium and low calcium diet in recurrent Swiss kidney stone formers

**DOI:** 10.1186/s12882-017-0755-7

**Published:** 2017-12-04

**Authors:** Harald Seeger, Andrea Kaelin, Pietro M. Ferraro, Damian Weber, Philippe Jaeger, Patrice Ambuehl, William G. Robertson, Robert Unwin, Carsten A. Wagner, Nilufar Mohebbi

**Affiliations:** 1 Division of Nephrology, University Hospital Zurich, Rämistr. 100, 8091 Zurich, Switzerland; 2 Division of Nephrology, Fondazione Policlinico Universitario A. Gemelli, Catholic University of the Sacred Heart, Rome, Italy; 3 Department of Urology, University Hospital Zurich, Zurich, Switzerland; 4 Centre for Nephrology, University College London, London, UK; 5 Division of Nephrology, Stadtspital Waid, Zurich, Switzerland; 6 Nuffield Department of Surgical Sciences, University of Oxford, Oxford, UK; 7 Institute of Physiology, University of Zurich, Zurich, Switzerland; 8 National Center for Competence in Research NCCR Kidney, CH, Zurich, Switzerland

**Keywords:** Nephrolithiasis, Urolithiasis, Hypercalciuria, Calcium oxalate, Diet

## Abstract

**Background:**

Kidney stone disease is common in industrialized countries. Recently, it has attracted growing attention, because of its significant association with adverse renal outcomes, including end stage renal disease. Calcium-containing kidney stones are frequent with high recurrence rates. While hypercalciuria is a well-known risk factor, restricted intake of animal protein and sodium, combined with normal dietary calcium, has been shown to be more effective in stone prevention compared with a low-calcium diet. Notably, the average sodium intake in Switzerland is twice as high as the WHO recommendation, while the intake of milk and dairy products is low.

**Methods:**

We retrospectively analyzed Swiss recurrent kidney stone formers (rKSF) to test the impact of a low-sodium in combination with a low-calcium diet on the urinary risk profile. In patients with recurrent calcium oxalate containing stones, we investigated both, the consequence of a low-sodium diet on urinary volume and calcium excretion, and the influence of a low-sodium low-calcium diet on urinary oxalate excretion.

**Results:**

Of the 169 patients with CaOx stones, 49 presented with hypercalciuria at baseline. The diet resulted in a highly significant reduction in 24-h urinary sodium and calcium excretion: from 201 ± 89 at baseline to 128 ± 88 mmol/d for sodium (*p* < 0.0001), and from 5.67 ± 3.01 to 4.06 ± 2.46 mmol/d (p < 0.0001) for calcium, respectively. Urine volume remained unchanged. Notably, no increase in oxalate excretion occurred on the restricted diet (0.39 ± 0.26 vs 0.39 ± 0.19 mmol/d, *p* = 0.277). Calculated Psf (probability of stone formation) values were only predictive for the risk of calcium phosphate stones.

**Conclusion:**

A diet low in sodium and calcium in recurrent calcium oxalate stone formers resulted in a significant reduction of urinary calcium excretion, but no change in urine volume. In this population with apparently low intake of dairy products, calcium restriction does not necessarily result in increased urinary oxalate excretion. However, based on previous studies, we recommend a normal dietary calcium intake to avoid a potential increase in urinary oxalate excretion and unfavorable effects on bone metabolism in hypercalciuric KSFs.

**Electronic supplementary material:**

The online version of this article (10.1186/s12882-017-0755-7) contains supplementary material, which is available to authorized users.

## Background

Kidney stones are common in industrialized countries with an increasing prevalence over the last several decades [1, 2]. Calcium-containing stones are the most frequent stone type and almost 40% of patients experience a second stone episode within 3 years of the first [3]. Importantly, several studies have described a significant association between kidney stones and adverse renal outcomes, including end stage renal disease (ESRD) [4–8], as well as an increased risk for coronary heart disease [9]. Thus, metaphylaxis plays a crucial role in treatment and has been shown to prevent stone recurrence and potentially chronic kidney disease [10], and may help to decrease the huge economic burden of stone disease, including expensive and repetitive urological interventions, large amounts of sick pay, and days lost from work, etc. [11]. While hypercalciuria is a well-known risk factor for kidney stone formation, restricted intake of animal protein and salt, combined with a normal calcium intake, has been shown to be more effective in the prevention of stone recurrence when compared with a low-calcium diet [12]. In particular, recurrent kidney stone formers (rKSFs) are recommended to reduce their salt intake, since high salt consumption itself is associated with hypercalciuria [13]. According to the last Swiss nutrition report, the average salt intake in Switzerland (10.6 g/d in males and 8.1 g/d in females) is approximately twice as high as the WHO recommendations (< 5 g/d). On the other hand, surprisingly, too little milk and dairy products are consumed (Sixth Swiss Nutrition Report 2012, www.blv.admin.ch), which could result in increased dietary oxalate absorption and urinary excretion. According to published data [12], a high sodium diet in combination with low dairy products and consequently low calcium intake in the Swiss population should lead overall to an increased dietary risk for kidney stone formation. However, other studies have shown a positive correlation between sodium excretion and 24-h urine volume, suggesting that sodium restriction may decrease thirst and thus fluid intake, resulting in an increased risk for kidney stone formation [14–16].

In our clinic, rKSFs were subjected to metabolic evaluation according to a standard protocol, including a first 24-h urine collection under normal diet, followed by a second 24-h urine collection after a seven-day low sodium and low calcium diet. In this study, we wanted to analyze the effect of a low sodium in combination with a low calcium diet on the urinary risk profile of recurrent calcium oxalate kidney stone formers. In particular, we wanted to determine both, the impact of a low sodium diet on urinary volume and calcium excretion, and the influence of a low calcium diet on urinary oxalate excretion.

## Methods

### Study population

Between February 2007 and July 2011, 154 male and 61 female patients who were referred to our stone clinic for recurrent kidney stone disease were included in this retrospective analysis. There were no exclusion criteria. For the analysis of the impact of the low calcium and low sodium diet on the urinary risk profile we only included patients with calcium oxalate containing stones (*n* = 169). The institutional review board of the University Hospital Zurich (Zentrum für klinische Forschung und Lehre) approved data collection and analyses.

At the first visit a detailed laboratory workup was performed, including blood and 24-h urine chemistry. After the first workup, patients undertook a calcium- and sodium-reduced diet for 7 days. The diet is routinely performed in our stone clinic to assess to which extent calciuria – one of the major risk factors of calcium oxalate stones - can be influenced by diet. On the last day of the diet we performed a second blood and 24-h urine analysis. Using a standardized protocol prepared by a dietician, the treating physician explained the diet to each patient. Patients were counselled to avoid any food products or beverages high in sodium and discouraged from adding table salt during the preparation of meals. In addition to sodium restriction, patients were instructed to avoid dairy products and mineral waters rich in calcium or calcium supplements during the seven-day period. In the case of full adherence, the diet provides an approximate daily intake of 346 mg calcium (range 211–480 mg or 5.3–12 mmol/d) and 1.25 g sodium (range 0.7–1.8 g or 31.8–78.8 mmol/d).

We used the Psf (probability of stone formation) algorithm for predicting stone risk and stone type that has been developed and validated by Robertson and colleagues [17, 18]. This calculation is based on a relative risk comparison of the key and routinely measured urinary stone risk and protective factors - urine volume, pH, calcium, oxalate, uric acid, citrate and magnesium - in controls and stone formers, and gives a probability score (typically in controls < 0.5 and in stone formers > 0.5) of stone formation and likely stone composition in the absence of any available stone analysis result. For the Psf calculations presented we combined patients with known stone composition in the above cohort (*n* = 192) with rKSF and known stone composition from a second cohort (*n* = 138) that was investigated in a separate study (Manuscript in preparation).

### Biochemical analyses

All laboratory analyses were performed at the Institute of Clinical Chemistry of the University Hospital Zurich. At baseline, 24-h urine was collected without addition of preservative agents or oil (native). The second 24-h urine collection was performed by adding thymol (1 g per 3 l urine container, Hänseler AG, Herisau, Switzerland) to inhibit bacterial growth and metabolization of urea to ammonia. In addition, mineral oil (75 ml per 3 l urine container, Hänseler AG, Herisau, Switzerland) was added to the second container when collection was started to allow for adequate pH measurement. Stone composition was determined by X-Ray diffractometry.

### Probability of stone formation (Psf)

As already described, the Psf value was calculated to test for predictability of the risk of formation: calcium oxalate, calcium phosphate, uric acid, or a combination of these stone types, and to assess whether the Psf might be influenced by the short-term dietary manipulation. For each patient with known stone composition, the Psf for stone formation was calculated for the following stone types: calcium oxalate, mixed calcium oxalate/phosphate, uric acid, mixed uric acid/calcium oxalate, and calcium phosphate; the closer the Psf value is to 1, the more likely it is that a patient will form a stone of that type. Patients with Psf values below 0.5 are deemed less likely to form stones and have a risk equivalent to that of non-stone formers.

### Definitions

Hypercalciuria was defined as urinary calcium excretion >6.25 mmol/day for female and >7.5 mmol/day for male subjects. Hyperoxaluria was defined as urinary oxalate excretion >0.5 mmol/day. Hyperuricosuria was defined as 24-h uric acid excretion >4.5 mmol/day for women and >4.8 mmol/day for men, and hypocitraturia as urinary citrate excretion <1.50 mmol/day. A stone was considered to be pure if it consisted of ≥95% of a single component.

### Statistical analyses

Demographic and clinical characteristics were documented for individual patients at the first visit. All statistical analyses were performed using SPSS Version 20.0 statistical package (SPSS Inc., USA) and Graphpad Prism Version 5 (GraphPad Software, Inc., USA). For paired analyses of normally distributed variables Student’s paired t-test (Shapiro-Wilks; *p* > 0.05) and for non-parametric data Wilcoxon test were used, respectively. Continuous variables were reported as means ± SD (standard deviation). Categorical variables were compared using the chi square test.

## Results

### Characteristics of study population

**Fig. 1:**
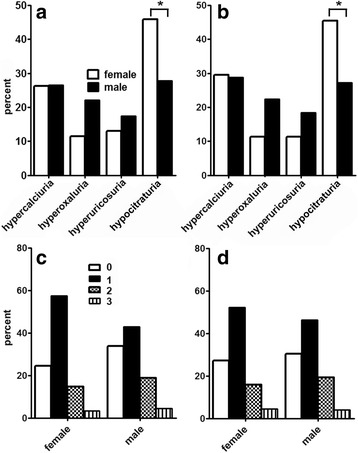
Frequency (**a, b**) and number (**c, d**) of metabolic risk factors in the whole cohort (**a, c**; *n* = 215) and in patients with calcium oxalate containing stones (**b, d**; *n* = 169) according to gender. The incidence of hypocitraturia at baseline was significantly higher in female patients (* *p* < 0.05.), whereas the other risk factors did not show a significant difference

Patient demographics are shown in Tables 1 and 2. Two hundred and fifteen patients were included in our study (mean age 47.1 ± 13.9 years), 154 were male and 61 females. As expected, pure calcium oxalate calculi were the most frequent stone type (42%). The second most common stone type was of mixed type, consisting of calcium oxalate combined with calcium phosphate or urate (38%). Calcium phosphate stones were significantly more common in female patients (8% vs. 2%, *p* = 0.043), whereas pure calcium oxalate stones were more frequent in men (46% vs. 31%, *p* = 0.048). Notably, stone composition was unknown in only 11% of all patients (Table 2). Figure 1a demonstrates urinary risk factors in all patients, while Fig. 1b displays risk factors of only patients with stones that were consisting entirely or partially of calcium oxalate. In our cohort, hypocitraturia was the most prevalent risk factor, followed by hypercalciuria (Fig. 1a). Notably, hypocitraturia was significantly more common in females (*p* = 0.013) (Fig. 1a, b), whereas hyperoxaluria and hyperuricosuria appeared to be more prevalent in male stone formers; however, the difference was not statistically significant. Hypercalciuria was slightly more common in patients with calcium oxalate containing stones (Fig. 1b) when compared with the whole cohort (Fig. 1a). Figure 1 (c, d) displays the prevalence of metabolic abnormalities in males and females. The majority of patients had one single metabolic abnormality (48%), whereas 18% had two, and 4% had a combination of three of them. In approximately 30% of all patients no urinary abnormality (hypercalciuria, hyperoxaluria, hyperuricosuria or hypocitraturia) was detectable (Fig. 1c). There was no significant difference in the number of urinary metabolic abnormalities between genders in the whole cohort (Fig. 1c) or in the subgroup of patients with calcium oxalate containing stones (Fig. 1d).

**Table 1:** Baseline characteristics of total cohort (standard deviation)

Characteristic	Male (*n* = 154)	Female (*n* = 61)	Total (n = 215)
Age, yr	46.9 (13.0)	47.6 (16.0)	47.1 (13.9)
Weight, kg	83.0 (14.3)	69.7 (16)	79.2 (16.0)
Body mass index, kg/m2	27.0 (3.9)	26.3 (6.2)	26.8 (4.6)
Number of stone episodes	3.4 (2.3)	2.8 (1.6)	3.2 (2.1)

**Table 2:** Stone composition by gender

Stone type	Male (*n* = 154)	Female (*n* = 61)	Total (*n* = 215)
Calciumoxalate n (%)	71 (46)	19 (31)	90 (42)
Apatite n (%)	3 (2)	5 (8)	8 (4)
Uric acid n (%)	6 (4)	1 (2)	7 (3)
Mixed n (%)	57 (37)	25 (41)	82 (38)
Others n (%)	3 (2)	2 (4)	5 (2)
Unknown n (%)	14 (9)	9 (14)	23 (11)

“Mixed” stones include Calciumoxalate with Apatite, Uric acid, Struvite, Brushite, or Whitlockite

### Effect of sodium and calcium reduced diet on urinary risk profile of patients with calcium oxalate stones

**Fig. 2:**
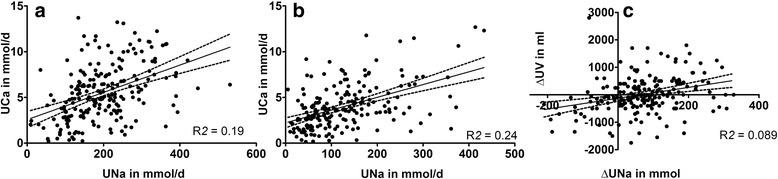
Correlation between 24-h urinary sodium and 24-h urinary calcium excretion pre- (**a**) and post-diet (**b**) and (**c**) between Δ 24-h urinary sodium and Δ 24-h urinary volume excretion in 215 recurrent kidney stone formers

One hundred and sixty-nine patients (125 males and 44 females) had calcium oxalate containing stones. Dietary reduction of sodium and calcium resulted in a significant reduction in 24-h urinary excretion of both sodium and calcium: 201 ± 89 at baseline vs. 128 ± 88 mmol/d for sodium (*p* < 0.001) and 5.67 ± 3.01 vs. 4.06 ± 2.46 mmol/d for calcium (p < 0.001) (Table 3). There was also a significant correlation between urinary sodium and calcium excretion (Fig. 2a, b). Despite an average 36% reduction of sodium excretion as a reflection of reduced intake, 24-h urine volume did not fall after 7 days of diet (2170 ± 904 vs. 2145 ± 724 ml/24 h) (Fig. 2c). In parallel with sodium and calcium, 24-h phosphate excretion significantly decreased after diet, probably reflecting the reduced intake of dairy and protein products (28.53 ± 10.52 vs. 24.04 ± 9.68 mmol/24 h, p < 0.001). In addition, an increase in plasma PTH was noted at the end of the low sodium low calcium diet probably in response to the decrease in calcium intake. Surprisingly, despite reduced calcium intake during the dietary period, no change in urinary oxalate excretion was found (0.39 ± 0.26 vs. 0.39 ± 0.19 mmol/d, *p* = 0.277). Additional file 1: Table S1 shows the effect of the low-sodium low-calcium diet on blood and urine parameters according to gender. Although male calcium oxalate stone formers showed a larger reduction in urinary sodium excretion after diet when compared with women, no decrease in urine volume could be observed at the end of the intervention. Furthermore, changes in blood and urine parameters were mostly similar in both male and female patients. We repeated the analysis after excluding the 12 patients with secondary causes of kidney stones. The results did not differ significantly in this subgroup (Additional file 2: Table S2).

**Table 3:** Plasma and urine chemistry in patients with calcium oxalate containing kidney stones at baseline and after seven days on low-calcium low-sodium diet

Blood parameters	Baseline (SD)	Diet (SD)
Creatinine in umol/l	86.3 (22.0)	88.3 (21.8)^*^
Sodium in mmol/l	141.4 (2.3)	141.6 (1.9)^ns^
Potassium in mmol/l	3.9 (0.3)	4.0 (0.4)^***^
Magnesium in mmol/l	0.82 (0.07)	0.84 (0.08)^***^
Bicarbonate in mmol/l	26.4 (2.5)	26.8 (2.7)^*^
Uric acid in mmol/l	331.4 (82.2)	351.1 (93.0)^***^
Urea in mmol/l	5.6 (1.9)	5.3 (2.0)^**^
Chloride in mmol/l	104.9 (2.7)	104.6 (2.8)^ns^
Calcium in mmol/l	2.3 (0.1)	2.3 (0.1)^ns^
Phosphate in mmol/l	1.0 (0.2)	0.9 (0.2)^ns^
iPTH in pg/l	47.4 (20.0)	49.2 (18.8)^*^
1,25-(OH)_2−_Vitamin D3 in ng/ml	52.0 (16.1)	nd
Urine parameters SD		
Volume in ml	2170 (904)	2145 (724)^ns^
Urinary pH	6.3 (0.6)	6.4 (0.6)^ns^
Sodium in mmol/d	200.7 (89.0)	128.3 (87.6)^***^
Potassium in mmol/d	65.8 (31.9)	60.7 (28.7)^*^
Chloride in mmol/d	195.0 (81.4)	128.0 (78.6)^***^
Calcium in mmol/d	5.7 (3.0)	4.1 (2.5)^***^
Magnesium in mmol/d	4.1 (1.7)	4.0 (1.8)^ns^
Phosphate in mmol/d	28.5 (10.5)	24.0 (9.7)^***^
Urea in mmol/d	416.3 (151.3)	366.7 (139.8)^***^
Creatinine in mmol/d	14.0 (4.4)	13.8 (4.7)^ns^
Uric acid in mmol/d	3.5 (1.3)	3.3 (1.2)^ns^
Glucose in mmol/d	4.0 (26.5)	2.3 (8.9)^ns^
Citrate in mmol/d	2.4 (1.6)	2.5 (1.5)^ns^
Oxalate in mmol/d	0.39 (0.26)	0.39 (0.19)^ns^
Ammonium in mmol/d	nd	42.2 (77.7)

*nd* not determined, *ns* non significant = *p* > 0.05, *p ≤ 0.05, **p ≤ 0.01, ***p ≤ 0.001; *SD* standard deviation

#### Adherence to low sodium and low calcium diet

**Fig. 3:**
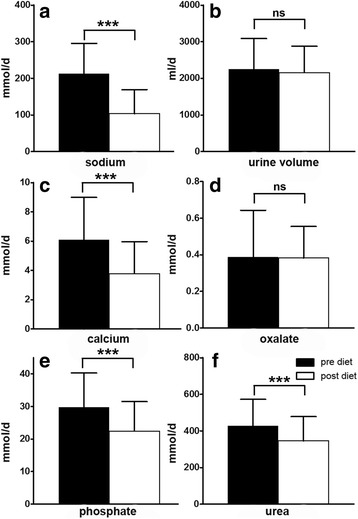
24 h excretion of sodium (**a**), urine volume (**b**), calcium (**c**), oxalate (**d**), phosphate (**e**) and urea (**f**) in calcium oxalate stone formers with successful dietary sodium restriction (mean (SD); ns = not significant = *p* > 0.05, *p ≤ 0.05, ** *p* ≤ 0.01, *** *p* ≤ 0.001; SD = standard deviation)

As described above, prescription of the 7-day-diet consisted of a standardized written protocol that was prepared by a dietitian and subsequently transmitted to the patient by the responsible physician. To minimize the effect of poor adherence to the diet on the results of urinary oxalate excretion and urine volume we performed a separate analysis in calcium oxalate stone formers after exclusion of all patients whose 24-h urinary sodium did not decrease while on diet. One hundred and thirty-one patients with calcium oxalate containing stones were included in this sub-analysis. On average, 24-h urinary sodium excretion decreased by 51% (212 ± 84 vs 104 ± 66 mmol/24 h, *p* < 0.001) in this group. In parallel, urinary calcium excretion declined from 6.07 ± 2.91 to 3.77 ± 2.19 mmol/24 h (38% reduction; p < 0.001). Again, no significant difference was observed for urine volume (2248 ± 836 vs. 2145 ± 724 ml/24 h; *p* = 0.081) or oxalate excretion in the 24 h urine collections (0.39 ± 0.26 before vs. 0.38 ± 0.17 mmol/24 h after diet, *p* = 0.607) (Fig. 3). However, 24-h excretion of phosphate (29.72 ± 10.60 vs. 22.42 ± 9.10 mmol/24 h (*p* < 0.001)) and urea (428 ± 144 vs. 346 ± 132 mmol/d (p < 0.001) were significantly reduced, indicating reduced dairy protein consumption. Interestingly, urinary magnesium excretion, considered as a protective factor, significantly decreased in patients that complied with the diet (4.3 ± 1.6 vs. 3.8 ± 1.7 mmol/24 h; *p* = 0.0001). Again, the results did not change significantly when we excluded the nine patients with secondary causes of nephrolithiasis (Additional file 3: Table S3).

## Effect of the sodium and calcium reduced diet on urinary risk profile of patients with hypercalciuria

Of 169 patients with calcium oxalate containing stones, 49 (29%) were hypercalciuric at baseline. After the 7-day combined low sodium and low calcium intervention, 12 patients remained hypercalciuric (9.91 ± 1.77 vs. 8.96 ± 1.99 mmol/24 h) (data were missing from 2 patients). In these patients, no significant reduction of urinary sodium excretion was detectable (243 ± 79 vs. 186 ± 123 mmol/24 h; *p* = 0.084), suggesting non-adherence to the prescribed dietary sodium restriction. In addition, phosphate excretion was not different (32.8 ± 9.9 vs. 29.7 ± 9.9 mmol/24 h; *p* = 0.388), indicating poor adherence to calcium reduction during the diet. In contrast, 35 of the hypercalciuric patients demonstrated a significant decrease in urinary sodium (235 ± 88 vs. 112 ± 83 mmol/24 h; *p* < 0.0001), and calcium excretion (9.24 ± 1.87 pre vs. 4.60 ± 1.44 mmol/24 h post diet; p < 0.001), respectively. In parallel, urinary phosphate excretion was significantly lower after the low sodium and low calcium dietary intervention (33.0 ± 8.4 vs. 25.0 ± 7.4 mmol/24 h; p < 0.0001). In contrast to previously published data, no increase in urinary oxalate excretion was observed (0.41 ± 0.14 vs. 0.41 ± 0.14, *p* = 0.437). In summary, in 75% of the patients with calcium oxalate nephrolithiasis and hypercalciuria, a short-term dietary intervention by reducing sodium and calcium in the diet resulted in a significant decrease in urinary calcium without a significant increase in urinary oxalate excretion. The above described results were not significantly different, when we excluded the three patients with secondary causes of nephrolithiasis (data not shown).

## Probability of stone formation (Psf)

We used the Psf score [17] to assess the predictability of stone composition in an extended cohort and to assess the effect of the 7-day low sodium and low calcium diet on Psf. In a cohort of 330 patients, including 215 patients described above (200 with CaOx, 22 with CaP, 13 with uric acid, 89 with mixed CaOx/CaP, and 6 with mixed uric acid/CaOx stones) the calculated Psf score from the baseline 24 h–urine parameters was only predictive with medium accuracy for CaP stones (Table 4a). In 215 patients that were subjected to the diet, the Psf for a CaP stone was significantly lower on diet, suggesting a decreased risk of developing calcium phosphate stones after dietary sodium and calcium restriction (Table 4b). However, in our patient population, the calculated Psf score according to other stone types did not correlate with stone composition.

**Table 4:** a) Probability of stone formation (Psf) in an extended cohort of 330 patients (median, interquartile range); b) Influence of low-salt low-calcium diet on Psf in the cohort of 215 patients with dietary intervention

a						
Factor	CaOx	CaP	UA	CaOx/CaP	UA/CaOx	*p*-value
N	200	22	13	89	6	
PSF CaOx,	0.25 (0.07, 0.68)	0.38 (0.10, 0.50)	0.03 (0.01, 0.21)	0.21 (0.08, 0.67)	0.13 (0.02, 0.17)	0.039
PSF CaP	0.5 (0.24, 0.72)	0.87 (0.75, 0.95)	0.23 (0.17, 0.32)	0.68 (0.45, 0.88)	0.28 (0.00, 0.49)	<0.001
PSF UA	0.00 (0.00, 0.00)	0.00 (0.00, 0.00)	0.00 (0.00, 0.00)	0.00 (0.00, 0.00)	0.00 (0.00, 0.00)	0.68
PSF CaOx/CaP,	0.33 (0.07, 0.73)	0.59 (0.24, 0.76)	0.10 (0.04, 0.32)	0.49 (0.12, 0.78)	0.08 (0.00, 0.16)	0.025
PSF UA/CaOx,	0.00 (0.00, 0.00)	0.00 (0.00, 0.00)	0.00 (0.00, 0.00)	0.00 (0.00, 0.00)	0.00 (0.00, 0.00)	0.73
b						
Stone type		Delta (95%, CI)			p-value	
CaOx		−0.06 (−0.10,-0.01)			0.02	
CaP		−0.09 (−0.13,-0.05)			<0.001	
Uric acid (UA)		0.02 (0.00,0.05)			0.06	
UA/CaOx		0.02 (−0.01,-0.05)			0.11	
CaOx/CaP		−0.06 (−0.11,-0.01)			0.03	

*CaOx* calcium oxalate, *CaP* calcium phosphate, *UA* uric acid, *CaOx/CaP* mixed calcium oxalate and calcium phosphate, *UA/CaOx* mixed uric acid and calcium oxalate, *CI* confidence interval

## Discussion

Hypercalciuria is the most frequent metabolic abnormality in individuals with calcium-containing kidney stones and can be found in up to 50% of these subjects [19, 20]. It can be caused by several conditions, including systemic diseases such as primary hyperparathyroidism or sarcoidosis, and if no underlying cause can be identified, it is considered idiopathic. Several mechanisms have been discussed in the pathogenesis of idiopathic hypercalciuria, such as increased intestinal calcium absorption and increased urinary calcium loss due to decreased proximal tubular reabsorption, and/or loss from bone [21, 22]. However, rather than a disease in itself, it probably reflects the upper end of the normal distribution of urinary calcium excretion in a population [23]. Nonetheless, the level of hypercalciuria is positively associated with the risk of developing calcium kidney stones [24]. In addition to an increase in fluid intake, which has been demonstrated to decrease the risk of recurrent kidney stones [25], reduction of sodium intake is another key strategy, since the excretion of sodium in urine strongly correlates with the magnitude of calciuria. Therefore, reduced sodium intake results in a reduction in urinary calcium excretion [26–31]. In our study, we could demonstrate that a short-term combined low sodium and low calcium dietary intervention also resulted in a significant reduction in urinary calcium excretion. In addition, other dietary factors with an effect on urinary calcium excretion such as the acid load from animal protein or ammonium chloride strongly increase urinary calcium excretion, whereas sodium bicarbonate administration mildly decreases calciuria [32]. Thus, the significant decline in calciuria in our patients may result from the combined effect of dietary sodium and calcium restriction, as well as from reduced consumption of animal proteins (dairy products) indicated by reduced phosphorus and urea excretion. Interestingly, citrate excretion and urine pH were unchanged before and after the diet, suggesting that the overall reduction of dietary protein intake was modest. However, since dietary calcium restriction has previously been shown to reduce calciuria only modestly [32], we assume that the impact of the short-term diet on calciuria was predominantly due to sodium restriction.

Hyperoxaluria is another important risk factor for calcium kidney stones [24]. Interestingly, the impact of dietary oxalate on urinary oxalate excretion and development of kidney stones appears to be small [33, 34], while the degree of enteric oxalate absorption seems to be more critical [35]. The latter is determined by different factors, including a significant intra- and inter-individual variability, and depends on the bioavailability of oxalate that can be affected by dietary intake of calcium or magnesium [35, 36]. In a randomized controlled trial by Borghi et al., a diet low in sodium and animal protein reduced stone recurrence rates after 5 years compared with a low calcium diet in male recurrent kidney stone formers with idiopathic hypercalciuria [12]. In both diet groups, urinary calcium excretion decreased by approximately 50%, similar to our hypercalciuric patients with calcium oxalate stones who complied with the low sodium diet. However, in the Borghi study, urinary oxalate excretion significantly increased in patients on a low calcium (10 mmol/day = 0.4 g/d) diet compared with patients on a combined low protein (93 g/day), low sodium (50 mmol/day) and normal calcium diet (30 mmol/day = 1.2 g/d), and remained high during the 5 years of follow-up. The increase in urinary oxalate was thought to be secondary to increased enteric absorption of oxalate when dietary calcium is low, and this was proposed as the underlying mechanism for the higher stone recurrence rate in this patient group. Previous interventional and observational studies have also described an inverse relationship between calcium (and animal protein) intake and risk of kidney stones [37–39]. These data have been confirmed by an interventional study which demonstrated that an increase in calcium intake led to a decrease in oxaluria within 1 week [40]. Thus, a low calcium diet is no longer recommended for the prevention of calcium oxalate stone recurrence [41]. Interestingly, the patients with calcium oxalate stones in our study did not show any increase in urinary oxalate excretion on a one-week low sodium and low calcium diet. There may be several reasons for this finding. First, a physician explained the diet to patients in addition to a written brochure, but there were no fixed or standardized meals and so the dietary calcium reduction might not have been sufficiently low to affect oxalate absorption and cause hyperoxaluria. The finding that plasma PTH increased significantly at the end of the diet supports, however, that dietary calcium intake was reduced. In addition, after exclusion of patients with unchanged sodium excretion - indicative of non-adherence to the diet - oxalate excretion was still not higher after 7 days on the low sodium and low calcium diet. Yet, even the adherent patients in our study probably did not lower their daily calcium intake to such low levels (150–250 mg/d) as in the above studies describing an increase in oxaluria upon dietary calcium reduction [37, 39]. Second, we cannot exclude that patients also reduced their oxalate intake while on the diet. However, as described earlier, the impact of dietary oxalate on oxaluria seems to be quite small [33] even though this assumption has been challenged by oxalate loading studies. Yet, oxalate loading studies should be interpreted cautiously, because of methodological problems regarding the reliability of the assays that have been used to measure oxalate, and it is also unclear whether the oxalate used in the oral loading studies has a similar bioavailability to oxalate in food [33, 35, 42–44]. Finally, by assuming that due to a low intake of dairy products (Sixth Swiss Nutrition Report 2012; www.blv.admin.ch) the average calcium intake in the Swiss population may already be low, a further reduction of calcium consumption may not have had any additional effect on enteric oxalate absorption. In a study by von Unruh and others, the authors found an inverse relationship between calcium intake and intestinal oxalate absorption [44]. A reduction of oral calcium intake by 70 mg/day was associated with a 1% increase in oxalate absorption. Consequently, if baseline calcium intake is already low (as assumed in our population), and absolute calcium reduction during the diet is only modest (e.g. from 700 to 400 mg/d), the effect of the dietary calcium reduction in our study on urinary oxalate excretion may be small [42]. In comparison with the studies by Borghi et al. [12] and Nakada et al. [40], our patients were also different with respect to other factors such as gender, ethnic background, and normal diet.

Magnesium excretion is positively associated with oxaluria, while decreases in sodium excretion or a reduced intake of animal protein appear to have no impact on hyperoxaluria [33]. The 7-day dietary intervention in our study led to a slight, but significant decrease in urinary magnesium excretion that may also explain the absence of any increase in oxalate excretion under the low sodium and low calcium diet.

Another important finding in our study is that even a significant reduction in sodium intake was not enough to affect thirst and to lower urine volume, since a low urine volume would be expected to increase stone risk. However, we cannot exclude that individuals coincidentally increased their fluid intake, e.g., following advice from their urologist, general practitioner, peers or recommendations found on the internet.

Lastly, in this particular Swiss population, the Psf score to predict stone risk and type was of limited value. This might be explained by genetic or environmental differences between the Swiss cohort and the population studied from which the algorithm was derived. Additionally, we performed the metabolic workup in most patients according to international guidelines weeks or months after the last stone episode or intervention, and stone analysis. Therefore, it is likely that a significant proportion of the patients had already changed their diet due to recommendations of the urologist or advice from other sources.

## Conclusion

Our data demonstrate that reducing sodium consumption in rKSFs in combination with a low calcium diet results in a significant reduction in urinary calcium excretion, but not a reduction in urine volume. Furthermore, calcium restriction in patients with low dietary calcium intake does not necessarily result in increased urinary oxalate excretion. Thus, a diet low in calcium, as currently in the wider Swiss population, seems to be safe in terms of the risk of stone recurrence.

To summarize, further prospective studies are required to test if the diet of the Swiss population is associated with a higher prevalence of hyperoxaluria and/or calcium oxalate kidney stone formation. However, based on previous studies, we believe that to avoid an increase in urinary oxalate excretion and unfavorable effects on bone metabolism, assessment and correction of low calcium intake in hypercalciuric KSFs is important and a normal dietary intake of calcium should be recommended.

## Additional files

Additional file 1: Table S1. Plasma and urine chemistry in female and male patients with calcium oxalate containing kidney stones at baseline and after seven days on low-calcium low-sodium diet. (DOCX 22 kb)

Additional file 2: Table S2. Plasma and urine chemistry in patients with calcium oxalate containing kidney stones at baseline and after seven days on low-calcium low-sodium diet. (DOCX 15 kb)

Additional file 3: Table S3. Plasma and urine chemistry at baseline and after seven days on diet from patients with calcium oxalate containing kidney stones which successfully reduced their sodium excretion on low-calcium low-sodium diet. (DOCX 16 kb)

## Abbreviations

CaOx: Calcium oxalate
CaP: Calcium phosphate
ESRD: End stage renal disease
Psf: Probability of stone formation
PTH: Parathyroid hormone
rSKSF: Recurrent Swiss kidney stone formers
WHO: World health organization
