# Supplementary material for: Changes in urinary risk profile after short-term low sodium and low calcium diet in recurrent Swiss kidney stone formers
Source: BMC Nephrol. 2017 Dec 4;18:349. doi: 10.1186/s12882-017-0755-7 (PMC5715611; doi:10.1186/s12882-017-0755-7)
Supplement: Supplementary file 1 — Plasma and urine chemistry in female and male patients with calcium oxalate containing kidney stones at baseline and after seven days on low-calcium low-sodium diet. (DOCX 22 kb) [file 12882_2017_755_MOESM1_ESM.docx]

|  | **male** | | | | | **female** | | | |
| --- | --- | --- | --- | --- | --- | --- | --- | --- | --- |
| **Blood parameters** | **baseline** | | | **diet** | | **baseline** | | **diet** | |
|  | **mean** | | **SD** | **mean** | **SD** | **mean** | **SD** | **mean** | **SD** |
| Creatinine in umol/l | 91.0 | | 20.8 | 91.8* | 16.6 | 72.9 | 19.8 | 77.8* | 30.5 |
| Sodium in mmol/l | 141.5 | | 2.3 | 141.8 | 1.9 | 141.0 | 2.3 | 140.8 | 1.9 |
| Potassium in mmol/l | 3.9 | | 0.3 | 4.0 | 0.4** | 3.9 | 0.3 | 4.0 | 0.4 |
| Magnesium in mmol/l | 0.82 | | 0.07 | 0.84** | 0.07 | 0.80 | 0.07 | 0.83** | 0.09 |
| Bicarbonate in mmol/l | 26.7 | | 2.4 | 27.2* | 2.6 | 25.6 | 2.5 | 25.8 | 2.7 |
| Uric acid in mmol/l | 354.2 | | 69.9 | 371.6** | 75.9 | 266.5 | 80.5 | 291.2** | 112.1 |
| Urea in mmol/l | 5.7 | | 1.7 | 5.3*** | 1.6 | 5.3 | 2.3 | 5.2 | 2.9 |
| Chloride in mmol/l | 104.6 | | 2.6 | 104.6 | 2.7 | 105.9 | 2.7 | 104.5** | 2.9 |
| Calcium in mmol/l | 2.4 | | 0.1 | 2.3 | 0.1 | 2.3 | 0.1 | 2.3 | 0.1 |
| Phosphate in mmol/l | 0.95 | | 0.17 | 0.92* | 0.16 | 0.99 | 0.16 | 0.99 | 0.18 |
| iPTH in pg/l | 46.8 | | 20.9 | 48.8 | 19.3 | 47.4 | 19.5 | 50.5 | 17.6 |
| 1,25-(OH)_2-_Vitamin D3 in ng/ml | 53.3 | | 16.5 | nd | nd | 48.4 | 14.8 | nd | nd |
|  |  | | |  | |  | |  | |
| **Urine parameters (SD)** |  | | |  | |  | |  | |
|  | **mean** | **SD** | | **mean** | **SD** | **mean** | **SD** | **mean** | **SD** |
| Volume in ml | 2208 | 867 | | 2157 | 706 | 2064 | 1003 | 2109 | 783 |
| Urinary pH | 6.3 | 0.6 | | 6.3 | 0.6 | 6.4 | 0.7 | 6.5 | 0.6 |
| Sodium in mmol/d | 219.4 | 82.1 | | 138.9*** | 93.6 | 148.0 | 87.4 | 96.0*** | 55.1 |
| Potassium in mmol/d | 70.2 | 33.1 | | 63.7* | 30.2 | 53.5 | 24.7 | 51.7 | 21.1 |
| Chloride in mmol/d | 214.2 | 77.6 | | 139.3*** | 82.4 | 138.8 | 65.3 | 91.8*** | 50.9 |
| Calcium in mmol/d | 6.0 | 3.0 | | 4.2*** | 2.6 | 4.7 | 2.9 | 3.7** | 2.1 |
| Magnesium in mmol/d | 4.4 | 1.6 | | 4.2 | 1.9 | 3.5 | 1.7 | 3.2 | 1.1 |
| Phosphate in mmol/d | 31.1 | 10.0 | | 26.1*** | 9.7 | 21.5 | 8.5 | 18.0** | 6.9 |
| Urea in mmol/d | 451.4 | 140.5 | | 393.1*** | 142.6 | 317.4 | 137.2 | 285.9 | 93.1 |
| Creatinine in mmol/d | 15.5 | 3.9 | | 15.0 | 4.6 | 9.8 | 3.2 | 10.0 | 2.2 |
| Uric acid in mmol/d | 3.7 | 1.1 | | 3.5 | 1.3 | 2.6 | 1.2 | 2.7 | 0.8 |
| Citrate in mmol/d | 2.5 | 1.5 | | 2.4 | 1.4 | 2.2 | 1.7 | 2.6 | 1.6 |
| Oxalate in mmol/d | 0.41 | 0.27 | | 0.42 | 0.20 | 0.33 | 0.21 | 0.32 | 0.14 |
| Ammonium in mmol/d | nd | nd | | 45.3 | 85.9 | nd | nd | 32.7 | 44.8 |

Additional file 1: **Table S1**. Plasma and urine chemistry in female and male patients with calcium oxalate containing kidney stones at baseline and after seven days on low-calcium low-sodium diet (nd = not determinded; *p ≤ 0.05. ** p ≤ 0.01. *** p ≤ 0.001; SD = standard deviation)
