# Supplementary material for: Changes in urinary risk profile after short-term low sodium and low calcium diet in recurrent Swiss kidney stone formers
Source: BMC Nephrol. 2017 Dec 4;18:349. doi: 10.1186/s12882-017-0755-7 (PMC5715611; doi:10.1186/s12882-017-0755-7)
Supplement: Supplementary file 2 — Plasma and urine chemistry in patients with calcium oxalate containing kidney stones at baseline and after seven days on low-calcium low-sodium diet. (DOCX 15 kb) [file 12882_2017_755_MOESM2_ESM.docx]

| **Blood parameters** | **baseline** | | **diet** | |
| --- | --- | --- | --- | --- |
|  | **mean** | **sd** | **mean** | **sd** |
| Creatinine in umol/l | 85.6 | 21.3 | 87.9 | 21.7* |
| Sodium in mmol/l | 141.4 | 2.2 | 141.6 | 1.9^ns^ |
| Potassium in mmol/l | 3.9 | 0.31 | 4.0 | 0.40*** |
| Magnesium in mmol/l | 0.82 | 0.07 | 0.84 | 0.08*** |
| Bicarbonate in mmol/l | 26.4 | 2.4 | 26.8 | 2.7* |
| Uric acid in mmol/l | 327.3 | 80.0 | 349.4 | 94.0*** |
| Urea in mmol/l | 5.5 | 1.9 | 5.3 | 2.0** |
| Chloride in mmol/l | 104.9 | 2.7 | 104.6 | 2.6^ns^ |
| Calcium in mmol/l | 2.3 | 0.1 | 2.3 | 0.1^ns^ |
| Phosphate in mmol/l | 1.0 | 0.2 | 0.93 | 0.16^ns^ |
| iPTH in pg/l | 46.7 | 19.8 | 48.7 | 17.9* |
| 1.25-(OH)_2-_Vitamin D3 in ng/ml | 51.2 | 15.5 | nd | |
|  |  | |  | |
| **Urine parameters** | **baseline** | | **diet** | |
|  | **mean** | **sd** | **mean** | **sd** |
| Volume in ml | 2195 | 874 | 2198 | 712^ns^ |
| Urinary pH | 6.3 | 0.6 | 6.4 | 0.6^ns^ |
| Sodium in mmol/d | 203.0 | 83.9 | 129.5 | 86.5*** |
| Potassium in mmol/d | 66.2 | 32.3 | 61.2 | 28.3^ns^ |
| Chloride in mmol/d | 197.8 | 78.0 | 128.4 | 77.3*** |
| Calcium in mmol/d | 5.7 | 3.0 | 4.2 | 2.5*** |
| Magnesium in mmol/d | 4.2 | 1.6 | 4.1 | 1.8^ns^ |
| Phosphate in mmol/d | 28.6 | 10.7 | 24.3 | 9.7*** |
| Urea in mmol/d | 421.1 | 146.8 | 372.5 | 139.8*** |
| Creatinine in mmol/d | 14.2 | 4.5 | 14.0 | 4.7^ns^ |
| Uric acid in mmol/d | 3.5 | 1.2 | 3.3 | 1.2^ns^ |
| Glucose in mmol/d | 2.1 | 9.4 | 2.3 | 9.2^ns^ |
| Citrate in mmol/d | 2.5 | 1.4 | 2.6 | 1.4^ns^ |
| Oxalate in mmol/d | 0.38 | 0.24 | 0.38 | 0.18^ns^ |
| Ammonium in mmol/d | nd | | 43.6 | 80.2 |

Additional file 2: **Table S2**. Plasma and urine chemistry in patients with calcium oxalate containing kidney stones at baseline and after seven days on low-calcium low-sodium diet (n = 157). Patients (n = 12) with secondary causes for kidney stone disease were excluded (n = 9 with bowel disease (n= 5 with Crohn’s disease in remission, n = 1 with celiac disease in remission, n = 3 stable condition post gastrectomy/gastric bypass), n = 2 with primary hyperparathyroidism (diagnosed later), n = 1 with primary hyperoxaluria (diagnosed later)). nd = not determined; ns = non significant = p > 0.05, *p ≤ 0.05, **p ≤ 0.01, ***p ≤ 0.001; SD = standard deviation.
