# Supplementary material for: Changes in urinary risk profile after short-term low sodium and low calcium diet in recurrent Swiss kidney stone formers
Source: BMC Nephrol. 2017 Dec 4;18:349. doi: 10.1186/s12882-017-0755-7 (PMC5715611; doi:10.1186/s12882-017-0755-7)
Supplement: Supplementary file 3 — Plasma and urine chemistry at baseline and after seven days on diet from patients with calcium oxalate containing kidney stones which successfully reduced their sodium excretion on low-calcium low-sodium diet. (DOCX 16 kb) [file 12882_2017_755_MOESM3_ESM.docx]

| **Blood parameters** | **baseline** | | **diet** | |
| --- | --- | --- | --- | --- |
|  | **mean** | **sd** | **mean** | **sd** |
| Creatinine in umol/l | 84.9 | 18.7 | 88.2 | 22.1** |
| Sodium in mmol/l | 141.5 | 1.9 | 141.4 | 1.9^ns^ |
| Potassium in mmol/l | 3.9 | 0.3 | 4.0 | 0.4*** |
| Magnesium in mmol/l | 0.82 | 0.07 | 0.85 | 0.08*** |
| Bicarbonate in mmol/l | 26.5 | 2.4 | 27.0 | 2.7* |
| Uric acid in mmol/l | 325.4 | 77.5 | 351.0 | 95.2*** |
| Urea in mmol/l | 5.5 | 1.9 | 5.2 | 2.1* |
| Chloride in mmol/l | 104.8 | 2.6 | 104.4 | 2.6^ns^ |
| Calcium in mmol/l | 2.3 | 0.1 | 2.3 | 0.1^ns^ |
| Phosphate in mmol/l | 0.96 | 0.16 | 0.94 | 0.16^ns^ |
| iPTH in pg/l | 44.8 | 17.3 | 48.5 | 17.0** |
| 1.25-(OH)_2-_Vitamin D3 in ng/ml | 52.4 | 15.6 |  | |
|  |  | |  | |
| **Urine parameters** | **baseline** | | **diet** | |
|  | **mean** | **sd** | **mean** | **sd** |
| Volume in ml | 2288.3 | 834.4 | 2208.2 | 703.7^ns^ |
| Urinary pH | 6.4 | 0.6 | 6.4 | 0.6^ns^ |
| Sodium in mmol/d | 214.4 | 82.2 | 105.4 | 63.4*** |
| Potassium in mmol/d | 68.6 | 33.9 | 59.3 | 28.4** |
| Chloride in mmol/d | 208.7 | 76.8 | 107.2 | 59.0*** |
| Calcium in mmol/d | 6.1 | 2.9 | 3.8 | 2.2*** |
| Magnesium in mmol/d | 4.4 | 1.6 | 3.9 | 1.7** |
| Phosphate in mmol/d | 30.1 | 10.8 | 22.8 | 9.1*** |
| Urea in mmol/d | 435.6 | 144.7 | 353.3 | 132.4*** |
| Creatinine in mmol/d | 14.4 | 4.4 | 13.6 | 4.5** |
| Uric acid in mmol/d | 3.6 | 1.2 | 3.1 | 1.1*** |
| Glucose in mmol/d | 2.3 | 10.3 | 2.0 | 9.3^ns^ |
| Citrate in mmol/d | 2.5 | 1.4 | 2.5 | 1.4^ns^ |
| Oxalate in mmol/d | 0.38 | 0.24 | 0.37 | 0.16^ns^ |
| Ammonium in mmol/d | nd | | 39.0 | 62.5 |

Additional file 3: **Table S3**. Plasma and urine chemistry at baseline and after seven days on diet from patients with calcium oxalate containing kidney stones which successfully reduced their sodium excretion on low-calcium low-sodium diet. Excluded where patients with secondary causes of nephrolithiasis (n = 9+) resulting in a total of 122 patients. ^+^n = 6 with bowel disease (n= 3 with Crohn’s disease in remission, n = 1 with celiac disease in remission, n = 2 stable condition post gastrectomy/gastric bypass), n = 2 with primary hyperparathyroidism (diagnosed later), n = 1 with primary hyperoxaluria (diagnosed later)). nd = not determined; ns = non significant = p > 0.05. *p ≤ 0.05. **p ≤ 0.01. ***p ≤ 0.001; sd = standard deviation.
